# Somatostatin-Mediated Regulation of Retinoic Acid-Induced Differentiation of SH-SY5Y Cells: Neurotransmitters Phenotype Characterization

**DOI:** 10.3390/biomedicines10020337

**Published:** 2022-02-01

**Authors:** Sneha Singh, Rishi K. Somvanshi, Ujendra Kumar

**Affiliations:** Faculty of Pharmaceutical Sciences, The University of British Columbia, Vancouver, BC V6T 1Z3, Canada; sneha901@student.ubc.ca (S.S.); rishiks@mail.ubc.ca (R.K.S.)

**Keywords:** neurite outgrowth, neurotransmitter, retinoic acid, SH-SY5Y cells, somatostatin

## Abstract

During brain development, neurite formation plays a critical role in neuronal communication and cognitive function. In the present study, we compared developmental changes in the expression of crucial markers that govern the functional activity of neurons, including somatostatin (SST), choline acetyltransferase (ChAT), tyrosine hydroxylase (TH), brain nitric oxide synthase (bNOS), gamma-aminobutyric acid (GABA), glutamic acid decarboxylase (GAD-65) and synaptic vesicle protein synaptophysin (SYP) in non-differentiated and retinoic acid (RA)-induced differentiated SH-SY5Y cells. We further determined the role of SST in regulating subcellular distribution and expression of neurotransmitters. Our results indicate that SST potentiates RA-induced differentiation of SH-SY5Y cells and involves regulating the subcellular distribution and expression of neurotransmitter markers and synaptophysin translocation to neurites in a time-dependent manner, anticipating the therapeutic implication of SST in neurodegeneration.

## 1. Introduction

The neurite formation, elongation, neurochemical composition, and integration are essential to establish a complex communicating neural network in the central nervous system (CNS). During embryonic development, the neural stem cells respond to the extrinsic factors and express distinct neurotransmitters and transcription factors in a specific temporal order to form a mature neuron [1,2]. The process of neural differentiation and maturation is tightly regulated by an interplay between neurotransmitters, morphogens, hormones, growth and trophic factors, as well as transcription factors [3,4]. To achieve such a diverse extrinsic cue for neurogenesis in an in vitro model is challenging due to the senescence of mature neurons. To cope with the challenges, previous studies have used the neuroblastoma cells (SH-SY5Y and pheochromocytoma 12 (PC12) cells) to induce differentiation from non-neuronal to the neuronal entity [5,6,7,8,9,10]. Upon addition of differentiating and/or trophic factors such as all-trans retinoic acid (RA), brain-derived neurotrophic factor, and nerve growth factor (NGF), the human-derived SH-SY5Y cells reproduce neurochemical, biochemical, and morphological properties of intact and functionally active neurons [5,6,9,11]. Previous studies suggest that different approaches of differentiation can influence specific neuronal subtypes such as adrenergic, cholinergic, and dopaminergic (DAergic) [11,12]. This property makes SH-SY5Y cells a valuable in vitro experimental tool to study neurological, specifically in neurotoxicological experiments [5,10,12,13,14]. RA, a vitamin A metabolite, is known to play a critical role in the development and maturation of the mammalian nervous system [15,16]. Studies have also reported that RA promotes survival of SH-SY5Y cells by activating the phosphoinositide 3-kinase/protein kinase B pathway and enhances choline acetyltransferase (ChAT), vesicular transport of monoamines, and tyrosine hydroxylase (TH) activity [5,13,17,18,19]. It has also been demonstrated that SH-SY5Y cells, in response to RA, differentiate to functional neurons with neurite formation and elongation [5,6,9,13]; however, the exact neurotransmitters phenotype of SH-SY5Y cells in undifferentiated and RA-induced differentiation is not well established.

The significant contribution of different neurotransmitters alone or in concert has been associated with neuronal differentiation, maturation, and dendrite growth to govern neuronal function. Amongst them, dopamine (DA), gamma-aminobutyric acid (GABA), serotonin, and glutamate play a crucial role. DA in CNS plays several fundamental roles, including neurological and neuropsychological diseases, behavioral locomotor function, attention deficit disorder, and addiction [20,21]. During differentiation, progenitor cells that are positive to TH have an ability to synthesize certain neurotransmitters, including ChAT, GABA, and substance P [22,23]. Although SH-SY5Y cells in response to RA have been reported for increased expression of TH, indicating prominent DAergic entity of differentiated cells [24], other studies contradict such observation and fail to see any change in TH expression [5]; however, neither morphological nor biochemical responses of TH in these cells are well understood. Similar to TH, other neurotransmitters, including brain nitric oxide synthase (bNOS) and acetylcholine (ACh), also govern the process of neuronal maturation and integration. Recent studies have highlighted the role of the cholinergic circuit during neural network development [25,26]. Nitric oxide (NO) has been studied extensively for its neurogenic response, especially during the proliferative stage of neurogenesis. On the other hand, studies also report that bNOS negatively regulates the process of neurogenesis [27]. Despite the extensive investigation on the role of these neurotransmitters in neurogenesis, there is a lacuna in understanding the pattern of subcellular distribution and expression of neurotransmitters and synaptic proteins during the developmental stages of neurite formation and maturation.

In neurological diseases, memory loss and impaired cognitive functions are associated with neuronal damage, perturbed synaptic formation, diminished dendrite arborization, neuronal network disruption, and neurochemical composition [28]. Somatostatin (SST), a well-studied neurotransmitter and neuromodulator, also plays a crucial role in neurite formation, elongation, and maturation [29,30,31]. Neuronal cells treated with SST displayed significant cell proliferation inhibition, indicating cell commitment into differentiated morphology [10,32]. It has been reported that depletion of SST affects the arborization of dendrites in the rat’s lateral superior olive, an auditory brainstem nucleus during early development [33], suggesting the trophic role of SST during neurogenesis and synaptogenesis. Growing evidence suggests that specific neuropeptides such as Neuropeptide Y and SST can promote neurite growth by activating G-protein coupled receptors (GPCRs) [10,31,34]. SST-mediated promotion of neurite outgrowth has also been observed in rat adrenal medulla-derived PC12 cells and cerebellar granule cells [35,36,37,38,39,40]. Thus, SST is an essential regulator of cell proliferation and differentiation [41]. In addition to regulating hormonal secretion, SST also regulates most, if not all, neurotransmitters in the CNS. Our recent findings showed that during the differentiation of SH-SY5Y cells, SST is associated with microtubules cytoskeleton protein [microtubule-associated protein 2 (MAP2), Tau and β-III-tubulin (TUJ1)] organization and regulates downstream signaling pathways to potentiate neurite formation [10]. Whether the changes seen in microtubules cytoskeleton proteins with SST treatment are also associated with the changes in neurotransmitters in RA-induced differentiated SH-SY5Y cells is still elusive. In the present study, we first aimed to determine the developmental changes in expression levels of neurotransmitter markers essential for neuronal integrity and functionality, including synaptophysin (SYP), SST, TH, bNOS, GABA, and glutamic acid decarboxylase (GAD-65) localization and expression levels following treatment with RA. Second, we determined the role of SST in combination with RA in the regulation of subcellular distribution and expression of neurotransmitter phenotype in undifferentiated and differentiated SH-SY5Y cells.

## 2. Materials and Methods

### 2.1. SH-SY5Y Cell Culture and Induction of Differentiation

Human SH-SY5Y neuroblastoma cells (Cat# 94030304; Sigma-Aldrich, Oakville, ON, Canada) were grown and maintained in Dulbecco’s modified eagle’s medium/Nutrient Mixture F-12 (DMEM/F-12; Invitrogen, Burlington, ON, Canada) supplemented with 10% fetal bovine serum (FBS) and 1% penicillin and streptomycin. Cells were maintained in a 5% CO_2_ humidified incubator at 37 °C as previously described [10,31]. To initiate RA-induced differentiation, cells were seeded on culture dishes pre-coated with Matrigel (10 mg/mL; BD Bio-science, San Jose, CA, USA) and grown for 1–7 days in a medium containing 2% FBS and RA (10 μM, Sigma; St. Louis, MO, USA). To examine the effect of SST (Bachem, Bubendorf, Switzerland), SH-SY5Y cells (control) were treated with SST (1 μM), RA (10 μM) alone, or in combination for 5 days. All experiments were carried out using cells within passages 14–22.

### 2.2. Cell Morphology and Quantitative Analysis of Neurite Outgrowth

Phase-contrast photomicrographs of SH-SY5Y cells were obtained using IncuCyte^TM^ live-cell imaging system (Essen Bioscience, Ann Arbor, MI, USA). Briefly, the cells were plated on a Matrigel pre-coated 12-well plate and were monitored for morphological changes with or without RA for 7-days (media was supplemented every 2 days). To explore the SST-mediated morphological changes, cells were either treated with SST alone or in combination with RA for 7-days. Images were captured every 3 h interval using a 10× objective. Multiple images of the SH-SY5Y cells collected post-treatment were analyzed for total neurite length as described by Pool et al. [42]. Briefly, we used the Neurite Tracer plugin of ImageJ software to detect the neurite length where the cell body was separated from neurites, and the neurites were traced to quantify their respective lengths as described previously [10]. For statistical analysis, approximately 100 cells were analyzed per image, and the average of the total neurite length per image was denoted as neurite length per cell. The D’Agostino–Pearson normality test was used to determine the normality of distribution in neurite length [43]. A two-way ANOVA test was performed using Graph Prism5.0 (GraphPad Inc, San Diego, CA, USA) for multiple comparisons.

### 2.3. Immunofluorescence Immunocytochemistry

SH-SY5Y cells were grown on Matrigel-coated glass coverslips in 24 well culture plates and treated with RA (10 µM) for 1, 3, 5, and 7 days. In parallel, to determine the effect of SST, cells were treated with RA with or without SST for 5 days. On the indicated day, cells were washed with phosphate-buffered saline (PBS, pH 7.4) and fixed using freshly prepared 4% paraformaldehyde for 15 min. Cells were then processed for immunostaining as previously described [10,31]. Briefly, the cells were blocked with 5% normal goat serum (NGS) in PBS for 1 h at room temperature (RT) and then incubated with primary antibodies including bNOS (1:250; Cat# ab3511; Abcam, Cambridge, UK), ChAT (1:600; Cat# AB144P; Millipore Sigma, Oakville, ON, Canada), GAD-65 (1:250; Cat# sc-377145), SST (1:300; Cat# sc-74556), SYP (1:100; Cat# sc-17750) (Santa Cruz Biotechnology, Santa Cruz, CA, USA), TH (1:100; Cat# 22941; ImmunoStar, Hudson, WI, USA) in 1% NGS overnight at 4˚C. The next day, the cells were washed in PBS followed by incubation with species-specific Cy3-conjugated secondary antibody (1:500; Jackson ImmunoResearch Laboratories, West Grove, PA, USA) in PBS for 2 h at RT. Following three subsequent washes in PBS, coverslips were mounted on the slides and photographed using Zeiss LSM700 confocal microscope (Carl Zeiss, Oberkochen, Germany). Immunocytochemical figures composites were constructed using Adobe Photoshop (Version 23.1, San Jose, CA, USA). The specificity of the immunoreactivity was validated in the absence of the primary antibody.

### 2.4. Western Blot Analysis

To analyze the expression of neurotransmitter markers by Western blot analysis, SH-SY5Y cells were grown in a 6-well plate pre-coated with Matrigel. Control and treated cells were washed in cold PBS and lysed with cold lysis buffer (Cat# 9803; Cell Signaling) containing 1% protease-phosphatase inhibitor mixture. The supernatant was collected, and protein concentration was determined using Bio-Rad protein assay (Bio-Rad Laboratories, Mississauga, ON, Canada). Whole-cell lysates (12 μg of total protein) were solubilized in Laemmli sample buffer containing 5% β-mercaptoethanol, heated for 5 min, and fractionated by SDS–PAGE, and transferred onto a nitrocellulose membrane. The membranes were blocked with 5% skim milk (Cat# sc-2324) in TBS-T (Tris-buffered saline with 0.05% Tween-20) for 1 h at RT and blotted overnight with bNOS (1:500), ChAT (1:1000), GAD65 (1:500), SST (1:600), SYP (1:600), and TH (1:500) specific primary antibodies. The membranes were washed with TBS-T and incubated for 2 h at RT with species-specific horseradish peroxidase (HRP)–conjugated secondary antibodies (1:1000; Jackson ImmunoResearch Laboratories) diluted using 5% skim milk in TBST. The membranes were washed and developed with an HRP-chemiluminescence substrate (Cat# WBLUR0500; Millipore, Billerica, MA, USA) and photographed using FluorChem 8800 software on the Alpha Innotech imaging system. β-actin (Cat# sc-47778 HRP; Santa Cruz Biotechnology) and vinculin (Cat# ab-91459; Abcam) (1:5000) were used as the loading control. Densitometric analysis was performed using FluorChem software (Alpha Innotech, San Jose, CA, USA).

### 2.5. Semi-Quantitative Analysis of Neurotransmitter Phenotype in Relative Cell Numbers Showing Developmental Changes

To further affirm the developmental changes in the neurotransmitter phenotype, we calculated the immunofluorescence intensity of non-differentiated and RA-induced differentiated SH-SY5Y cells using ImageJ as described previously [44]. The calculated immunoreactivity was assigned with an intensity range (0–195), where 0 represents the darkest shade and 195 represents the lightest shade. Post analysis, immunofluorescence intensity was grouped as follows: measurements between 40 and 90 were considered as strongly positive (+++); 91–140 were considered as moderately positive (++); 141–195 represents weakly positive immunostaining (+).

### 2.6. Statistical Analysis

The results are derived from three independent experiments and presented as mean ± standard deviation (SD). All statistical analysis was performed by using Graph Prism5.0 (GraphPad Inc, San Diego, CA, USA). One-way or two-way analysis of variance (ANOVA) followed by Tukey’s post hoc analysis was used for the analysis of statistical significance and * *p* < 0.05 against control, or # *p* < 0.05 within the pair were taken into consideration as significant.

## 3. Results

### 3.1. Retinoic Acid-Induced Morphological Changes, Neurite Formation, and Elongation of SH-SY5Y Cells

**Figure 1:**
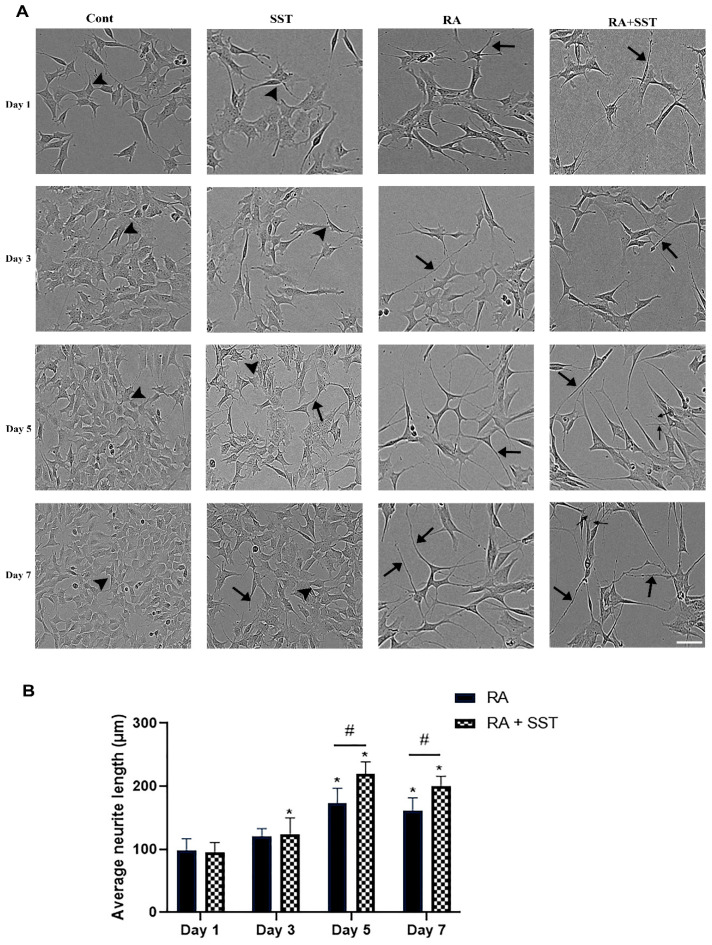
(**A**) Representative phase-contrast photographs displaying comparative time-dependent morphological changes in control and cells treated with SST, RA, and SST + RA. Control (undifferentiated) SH-SY5Y cells displayed extensive cell proliferation and grew in a cluster with a non-polarized cell body (arrowheads). SST alone promoted neurite formation but less than RA; SST potentiated RA-induced differentiation with elongated neurites outgrowth (arrows) and dendrites arborization (thin arrows). Scale bar = 100 µm. (**B**) SST promotes retinoic acid-induced neurite formation in SH-SY5Y cells. SH-SY5Y cells were treated with RA (10 µM) alone and/or with SST (1 µM) for days 1–7, as described in the experimental section. On the indicated days of experiments, neurite growth was quantified using Image J. Note a significant increase in neurite length in the cells treated with RA + SST at days 5 and 7 compared to RA alone. Results are presented as mean ± SD (n = 3). * p < 0.05 against respective Day1 and # *p* < 0.05 within the pair.

We first evaluated the time-dependent morphological changes and the neurites formation in cells treated with RA, SST, and RA + SST for 1 to 7 days. As shown in Figure 1A, compared to non-differentiated cells, the cell treated with RA showed significant promotion of the neurite formation, displaying the commitment of RA to the differentiation process. Cells displaying neurite formation are often elongated and spindle shape, whereas the cells devoid of neurites were relatively large in size and displayed flat morphology (Figure 1A). SH-SY5Y cells treated with SST alone also prompt the neurite formation; however, neurite elongation was less prominent than RA alone. Neurite formation and elongation were significantly increased in cells treated with SST along with RA compared to RA or SST alone. Importantly, we also observed extensive dendritic arborization when SST was used in combination with RA (Figure 1A).

Next, we quantified the average length of neurite outgrowth at days 1, 3, 5, and 7 of differentiation with RA alone and in combination with SST. The neurite length was consistently increased with increasing days of differentiation (Figure 1B). In the presence of SST + RA, SH-SY5Y cells displayed enhanced neurite outgrowth compared to RA alone. Cells in response to RA displayed neurite formation with an average length of 98 ± 18 μm, 120 ± 12 μm, 172 ± 21 μm, and 160 ± 19 μm as compared to RA + SST with an average length of 95 ± 14 μm, 124 ± 21 μm, 222 ± 16 μm and 200 ± 12 μm on days 1–7, respectively. Taken together, these morphological changes revealed that SST significantly promotes RA-induced differentiation to the neuronal entity with changes in neurites formation in SH-SY5Y cells. Notably, the neurite length was not significantly different on days 5 and 7, suggesting a plateau of neurite outgrowth and terminal differentiation of SH-SY5Y cells by day 5.

**Figure 2:**
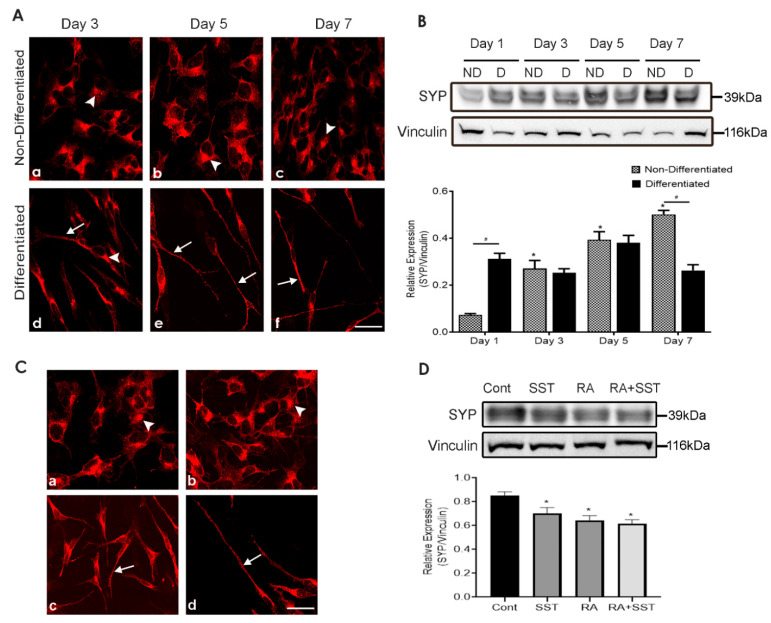
Time-dependent and SST-mediated changes in SYP localization in undifferentiated and differentiated SH-SY5Y cells. (**A**) SYP immunoreactivity was confined to the perinuclear region, possibly in the *trans*-Golgi network of undifferentiated cells (**a**–**c**). Note the reduced number of cells displaying localization in *trans*-Golgi network with RA treatment and strong localization of SYP in neurites (**d**–**f**). (**B**) Western blot analysis of SYP in undifferentiated and differentiation cells. The histogram represents densitometric analysis of relative expression of SYP. * *p* < 0.05 against respective day 1, # *p* < 0.05 within the pair. (**C**) In comparison to control (**a**), cells treated with SST (**b**), RA (**c**), and RA + SST (**d**) displayed SYP immunoreactivity in neurites and reduced expression in the perinuclear region. (**D**) Immunoblot analysis displayed decreased expression levels of SYP upon treatment, as indicated. Vinculin was used as a loading control. Arrowheads and arrows indicate immunoreactivity in the cell body and neurites, respectively. Results are presented as mean ± SD (n = 3). * *p* < 0.05 against control. Scale bar = 20 μm.

**Figure 3:**
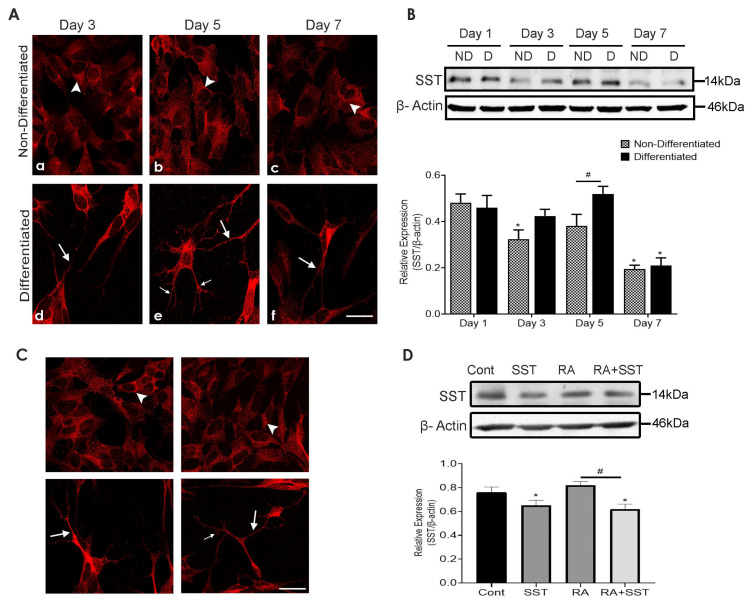
Subcellular distribution and expression of SST in SH-SY5Y cells. (**A**) Undifferentiated SH-SY5Y cells exhibited mild SST immunoreactivity at early time points (**a** and **b**), which was reduced on day 7 (**c**). In response to RA, cells displayed neurite formation, and neurites bearing cells showed strong SST expression with reduced expression by day 7 (**d**–**f**). (**B**) Immunoblot analysis showing SST expression on days 1–7. Histogram represents densitometric analysis of relative expression of SST. * *p* < 0.05 against respective day 1, # *p* < 0.05 within the pair. (**C**) SST-like immunoreactivity (**a**–**d**) and expression levels (**D**) were suppressed in response to SST alone and in combination with RA when compared to control and RA only. The histogram represents densitometric analysis of relative expression of SST. β-actin was used as a loading control. Arrowheads and arrows indicate immunoreactivity in the cell body and neurites, whereas thin arrows indicate dendritic arborization. Results are presented as mean ± SD (n = 3). * *p* < 0.05 against control, # *p* < 0.05 when compared to RA. Scale bar = 20 μm.

**Figure 4:**
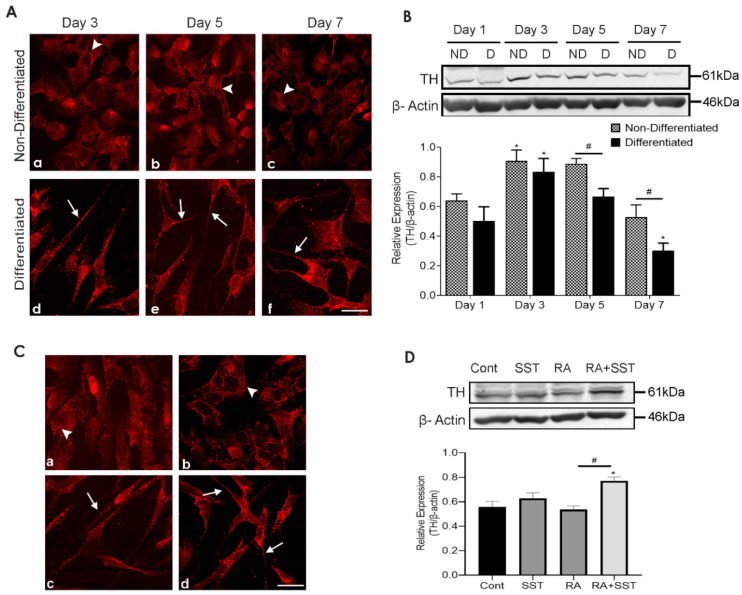
Time-dependent and SST-mediated changes in subcellular distribution and expression of TH in SH-SY5Y cells**.** (**A**) Undifferentiated SH-SY5Y cells displayed mild punctuated TH staining at days 3 and 5 (**a**,**b**) when compared to day 7 (**c**) In differentiated cells, enhanced TH immunoreactivity was seen in neurites as well as intracellularly with reduced expression at day 7 (**d**–**f**). (**B**) Representative immunoblot displaying the levels of TH in control and treated cells. The histogram represents densitometric analysis of relative expression levels of TH. * *p* < 0.05 against respective day 1, # *p* < 0.05 within the pair. (**C**) Confocal images of TH (**a**–**d**) and immunoblot (**D**) showed increased immunoreactivity and expression levels of TH with RA + SST treatment when compared to control and RA alone. β-actin was used as a loading control. Arrowheads and arrows indicate immunoreactivity in the cell body and neurites, respectively. Results are presented as mean ± SD (n = 3). * *p* < 0.05 against control, # *p* < 0.05 when compared to RA. Scale bar = 20 μm.

**Figure 5:**
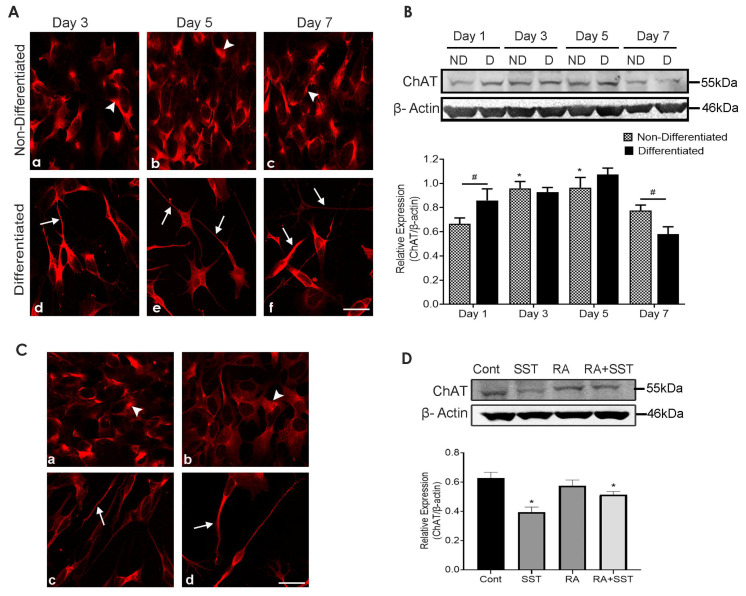
Immunocytochemical localization and immunoblot analysis showing changes in ChAT in SH-SY5Y cells. (**A**) Undifferentiated SH-SY5Y cells displayed ChAT staining in the cell body with no significant changes on days 3 and 5; however, reduced on day 7 (**a**–**c**). ChAT immunoreactivity was confined to the cell body and neurites and increased in the presence of RA till day 5 and suppressed on day 7 (**d**–**f**). (**B**) Representative Western blot analysis showing developmental expression of ChAT levels at the respective days. Histogram represents densitometric analysis of relative expression of ChAT. * *p* < 0.05 against respective day 1, # *p* < 0.05 within the pair. (**C**) ChAT immunoreactivity (**a**–**d**) and expression levels (**D**) were decreased following treatment with SST and SST + RA, whereas they remained unchanged in the presence of RA when compared to control. The histogram represents densitometric analysis of relative expression of ChAT. β-actin was used as a loading control. Arrowheads and arrows indicate immunoreactivity in the cell body and neurites, respectively. Results are presented as mean ± SD (n = 3). * *p* < 0.05 against control. Scale bar = 20 μm.

**Figure 6:**
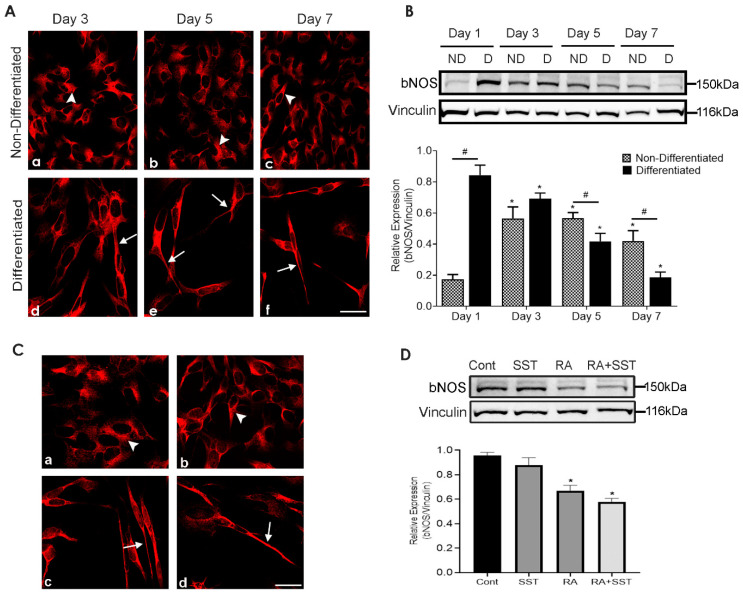
bNOS expression is decreased in response to RA treatment. (**A**) strong cytoplasmic expression of bNOS in undifferentiated SH-SY5Y cells (**a**–**c**) compared to strong immunoreactivity in neurite formation when cells exposed to RA (**d**–**f**). (**B**) SH-SY5Y immunoblot displaying the opposing trend of bNOS expression with and without RA treatment in a time-dependent manner. Histogram represents densitometric analysis of relative expression of bNOS. * *p* < 0.05 against respective day 1, # *p* < 0.05 within the pair. (**C**) bNOS-like immunoreactivity (**a**–**d**) and expression levels (**D**) were decreased in cells treated with RA, and RA + SST Vinculin was used as a loading control. Arrowheads and arrows indicate immunoreactivity in the cell body and neurites, respectively. Results are presented as mean ± SD (n = 3). * *p* < 0.05 against control. Scale bar = 20 μm.

**Figure 7:**
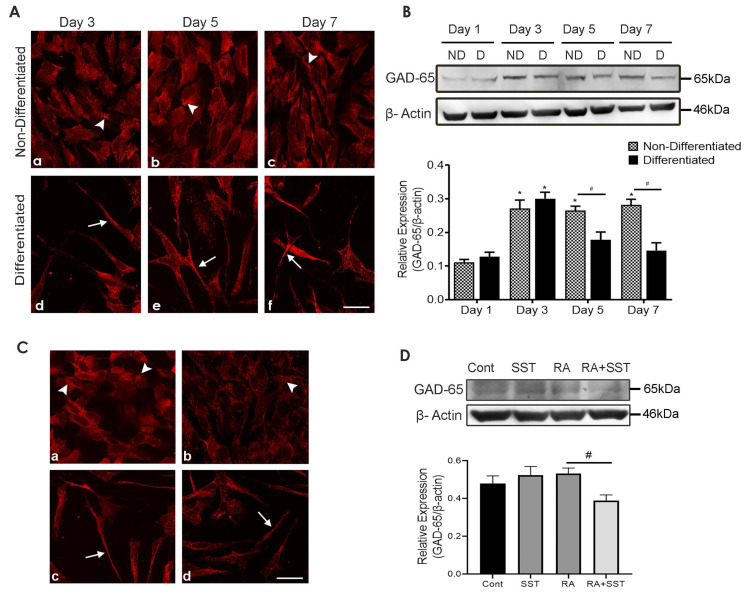
Transient increase in GAD expression in response to RA. (**A**) GAD-65-like immunoreactivity was expressed in the cell body of undifferentiated (**a**–**c**) and in neurites of RA treated cells (**d**–**f**). (**B**) Western blotting of GAD-65 in undifferentiated and differentiated SH-SY5Y cells. Histogram represents densitometric analysis of relative expression of GAD-65. * *p* < 0.05 against respective day 1, # *p* < 0.05 within the pair. (**C**) The subcellular distribution of GAD-65 (**a**–**d**) and immunoblot (**D**) showed decreased expression in cells treated with RA + SST. Histogram represents densitometric analysis of relative expression of GAD-65. β-actin was used as a loading control. Arrowheads and arrows indicate immunoreactivity in the cell body and neurites, respectively. Results are presented as mean ± SD (n = 3). * *p* < 0.05 against control. Scale bar = 20 μm.

During the course of differentiation of SH-SY5Y cells from non-neuronal to the neuronal entity, multiple neurotransmitters and neuropeptides are involved in a well-defined and articulated manner [9]. Accordingly, we analyzed the developmental changes in neurochemical markers as an index of neuronal characteristics and functionality. Subcellular distribution and expression were determined using immunofluorescence immunocytochemistry on days 3, 5, and 7, whereas quantitative analysis of immunoreactivity was accomplished using Western blot analysis. SST plays a crucial role in synthesizing and releasing several neurotransmitters in the CNS [45]. To address if SST regulates neurotransmitters during differentiation, we next treated the undifferentiated (control) SH-SY5Y cells with SST (1 μM), RA (10 μM), and SST + RA for 5 days, and post-treatment cells were processed for immunocytochemistry and Western blot analysis. The results of developmental changes are presented in Figure 2, Figure 3, Figure 4, Figure 5, Figure 6 and Figure 7 (panels A and B), S1, and Table 1, whereas Figure 2, Figure 3, Figure 4, Figure 5, Figure 6 and Figure 7 (panels C and D) depict the effects of SST with/without RA.

### 3.2. Developmental Changes in Synaptophysin in SH-SY5Y Cells

SYP is one of the markers for neuronal maturity and synapse formation. To attest whether differentiated SH-SY5Y cells are functionally active, the subcellular distribution and expression level of SYP was determined. In undifferentiated cells, strong SYP immunoreactivity in the form of clusters was seen mainly confined to the perinuclear region, probably in the *trans*-Golgi network (TGN) (Figure 2A, panels a–c). In comparison, while showing gradual neurite outgrowth in response to RA, differentiated cells displayed SYP at the apical endings and neurites (Figure 2A, panels d–f). Interestingly, SYP-like immunoreactivity was gradually decreased in perinuclear regions while translocating to the apical endings and neurites in a time-dependent manner upon treatment with RA (Figure 2A, panels d–f).

We further extended our study and determined the expression of SYP by Western blot analysis (Figure 2B). SYP expression gradually increased in undifferentiated cells. An augmented expression level of SYP was observed in response to RA. Interestingly, compared to undifferentiated SH-SY5Y cells, SYP expression was significantly higher at day 1 in the presence of RA but reduced at day 3, albeit non-significantly, and peaked at day 5. On day 7 of differentiation, the relative expression of SYP was significantly less than non-differentiated cells.

### 3.3. SST Promote Synaptophysin Translocation to Neurites Formation

We next sought to determine the dynamic response of SYP upon treatment with SST alone and in combination with RA. In non-differentiated SH-SY5Y cells, SYP-like immunoreactivity was mainly confined to the perinuclear regions, probably in the TGN (Figure 2C, panel a). Undifferentiated SH-SY5Y cells exposed to SST displayed SYP localized translocation to the apical ending of cells as well as to cytoplasm (Figure 2C, panel b); however, when cells were treated with RA alone and in combination with SST, cells exhibited suppressed SYP-like immunoreactivity in the perinuclear region and a strong immunoreactivity in neurites (Figure 2C, panels c and d). Western blot analysis showed comparable results as immunocytochemistry; however, immunoblot analysis showed decreased SYP expression when cells were treated with SST, RA alone, and RA + SST compared to control (Figure 2D).

### 3.4. Time-Dependent Changes in Subcellular Distribution and Expression of Somatostatin

The subcellular distribution of SST-like immunoreactivity in SH-SY5Y cells exhibited a distinct distribution pattern in undifferentiated vs. differentiated cells. As shown in Figure 3A, undifferentiated SH-SY5Y cells exhibited mild but comparable SST immunoreactivity at days 3 and 5 (Figure 3A, panels a and b), whereas SST staining was suppressed on day 7 (Figure 3A, panel c). Compared to undifferentiated SH-SY5Y cells, RA treated cells (Figure 3A, panels d–f) displayed neurites formation, and neurites bearing cells displayed strong SST expression in the cell body and neurite growth and dendrites arborization (Figure 3A, panel e).

Cell lysate prepared from undifferentiated and RA-induced differentiated cells was immunoblotted with SST antibody. Undifferentiated and RA-treated cells exhibited comparable SST immunoreactivity at day 1 (Figure 3B). In undifferentiated SH-SY5Y cells, SST expression was highest on day 1, followed by suppression on days 3 and 5, with the least expression on day 7. Differentiated SH-SY5Y cells showed comparable SST expression between days 1, 3, and 5, whereas, at day 7, SST expression was reduced significantly and was comparable to day 7 of undifferentiated cells. Taking together these results suggest that total SST immunoreactivity in SH-SY5Y cells is not significantly changed in differentiated cells between days 1 to 5 but decreases significantly at day 7. Undifferentiated SH-SY5Y cells exhibit a bimodal pattern of expression (Figure 3B).

### 3.5. Somatostatin Regulates Its Own Expression in SH-SY5Y Cells

It is well established that SST inhibits its release through negative feedback [46]. To examine what role SST might play in SH-SY5Y cells during RA-induced differentiation, SST expression was determined by immunocytochemistry and immunoblot analysis. As shown in Figure 3C, SST-like immunoreactivity in non-differentiated SH-SY5Y cells was distributed throughout the cytoplasm (Figure 3C, panel a). Selected neurites bearing cells displayed strong SST staining in response to SST treatment compared to non-differentiated cells (Figure 3C, panel b). Following treatment with RA, SST staining was confined to the apical ending of the cells and the extended neurites outgrowths (Figure 3C, panel c); however, SST immunoreactivity in the cells treated with SST + RA was translocated to the neurite formation in granular structures, probably synaptic vesicles (Figure 3C, panel d).

SST immunoreactivity was quantified using immunoblot analysis (Figure 3D). In comparison to undifferentiated SH-SY5Y cells, reduced SST expression was observed in cells treated with SST alone. In the presence of RA, SST expression was non-significantly higher than undifferentiated SH-SY5Y cells, which were further reduced upon treatment with SST + RA. Taken into consideration, these results suggest that SST is involved in its own expression and probably initiates its release from cells to the culture medium.

### 3.6. Time-Dependent Changes in Tyrosine Hydroxylase Immunoreactivity in Undifferentiated and Differentiating SH-SY5Y Cells

TH-like immunoreactivity in SH-SY5Y cells in the absence or upon RA treatment displayed a distinct subcellular distributional pattern intracellularly. As shown in Figure 4A, undifferentiated SH-SY5Y cells exhibited mild TH immunoreactivity at day 3 (Figure 4A, panel a) compared to differentiated cells displaying TH immunoreactivity in the cytoplasm and neurites (Figure 4A, panels d). On day 5, the relative expression of TH-like immunoreactivity was enhanced along with punctate granular staining at the cell surface in the absence of RA (Figure 4A, panel b). When compared to days 3 and 5, TH immunoreactivity in non-differentiated cells decreased on day 7 (Figure 4A, panel c). Moreover, TH immunostaining in differentiated cells was enhanced in neurite formation as well as intracellularly on days 3 and 5 (Figure 4A, panels d and e). It was interesting to note that at day 7 of differentiation, TH immunostaining was relatively less in cells devoid of neurites than the neurite-bearing differentiated cells (Figure 4A, panel f).

TH expression determined using Western blot analysis revealed comparable expression on days 1 and 3 between non-differentiated and RA treated SH-SY5Y cells; however, TH expression was significantly higher on day 3 in comparison to day 1. On days 5 and 7, TH expression was significantly lower in differentiated cells compared to undifferentiated cells (Figure 4B). These results confirm that TH immunoreactivity exists in undifferentiated and differentiated SH-SY5Y cells with the highest expression on day 3.

### 3.7. Somatostatin-Mediated Regulation of TH Expression in SH-SY5Y Cells

The reciprocal regulation of DA and SST synthesis and release is well established in selective brain regions. TH-like immunoreactivity in non-differentiated control cells was largely confined intracellularly in a variable intensity (Figure 4C, panel a). TH-like immunoreactivity was enhanced in cells treated with SST, and a granular pattern of TH distribution was evidenced at the cell surface as well as intracellularly in SH-SY5Y cells (Figure 4C, panel b). The intracellular TH localization was suppressed with RA treatment, and cells displayed TH expression at the cell surface and neurites (Figure 4C, panel c). The cells treated with SST in combination with RA exhibited strong TH expression in the cell body and neurites compared to SST or RA treatment alone (Figure 4C, panel d). Interestingly, cells treated with RA + SST exhibited elongated neurite formation, and cells with leading neurite outgrowth displayed strong TH expression.

Immunoblot analysis revealed insignificantly increased TH immunoreactivity when SH-SY5Y cells were treated with SST compared to control and RA-treated cells. In addition, enhanced TH expression was seen in cells treated with SST along with RA, greater than RA alone (Figure 4D).

### 3.8. Developmental Changes in Choline Acetyltransferase in SH-SY5Y Cells

Undifferentiated SH-SY5Y cells displayed cytoplasmic ChAT staining with no significant variation at days 3 and 5 (Figure 5A, panels a and b) but reduced staining at day 7 (Figure 5A, panel c). In contrast, when cells were treated with RA for days 3, 5, and 7, ChAT-like immunoreactivity was present intracellularly and along with neurite formations (Figure 5A, panels d–f). Furthermore, ChAT immunoreactivity in differentiated cells was gradually enhanced in a time-dependent manner till day 5 and suppressed at day 7 (Figure 5A, panels e and f).

In parallel to immunocytochemistry, similar results were seen by Western blot analysis (Figure 5B). ChAT expression was significantly higher in differentiated cells than undifferentiated cells at day 1 and remained comparable on day 3; however, ChAT levels in undifferentiated cells at day 3 were significantly higher in comparison to day 1. Immunoblot analysis revealed no discernable difference between differentiated or undifferentiated cells on day 5; however, on day 7, ChAT expression was decreased in both undifferentiated and differentiated SH-SY5Y cells. Notably, ChAT expression was significantly lower in differentiated cells when compared to undifferentiated SH-SY5Y cells on day 7 (Figure 5B).

### 3.9. Somatostatin Decreases ChAT Expression in SH-SY5Y Cells

Immunocytochemical and Western blot analysis of undifferentiated and differentiated SH-SY5Y cells showed a widespread distribution of ChAT-like immunoreactivity and changes in expression following treatment as indicated. Control undifferentiated cells displayed strong ChAT-like immunoreactivity (Figure 5C, panel a) compared to cells treated with SST alone (Figure 5C, panel b). ChAT-like immunoreactivity was not changed upon RA treatment; rather, strong expression was seen in neurites formation (Figure 5C, panel c). In contrast, strong ChAT immunoreactivity was observed in the neurite formation in differentiated cells treated with SST (Figure 5C, panel d).

As shown in Figure 5D, consistent with subcellular expression, immunoblot analysis revealed suppressed ChAT expression in cells treated with SST compared to control cells. Cells treated with RA alone exhibited no significant changes compared to control SH-SY5Y cells, whereas, in cells treated with SST + RA, ChAT expression was significantly reduced in comparison to control.

### 3.10. Time-Dependent Changes in Brain Nitric Oxide Synthase Immunoreactivity in SH-SY5Y Cells

To determine whether bNOS is expressed in SH-SY5Y cells and involved in neurite outgrowth following RA-induced differentiation, bNOS expression levels were determined on respective days as indicated using immunocytochemistry and immunoblot analysis. The comparative subcellular localization of bNOS at days 3–7 in non-differentiated cells showed no distinguishable changes (Figure 6A, panels a–c); however, upon treatment with RA, cells exhibited a strong bNOS staining in comparison to undifferentiated SH-SY5Y cells (Figure 6A, panels d–f). As shown in Figure 6A, moderate bNOS-like immunoreactivity was observed in the neurites of differentiated cells, without any noticeable changes from days 3–7 (Figure 6A, panels d–f)

Concomitant to our immunostaining, similar results were obtained using Western blot analysis. The quantitative analysis of bNOS expression revealed an opposite trend of bNOS expression with and without RA treatment in a time-dependent manner (Figure 6B). In undifferentiated SH-SY5Y cells, bNOS expression was increased significantly at days 3, 5 and 7 compared to day 1 without any significant variation at days 3 and 5. In contrast, cells treated with RA exhibited an almost 5-fold increase in bNOS expression at day 1, and bNOS expression level was gradually declined in a time-dependent manner from day 3 to 7, respectively (Figure 6B). On day 7, bNOS expression in differentiated cells was significantly lower than undifferentiated cells.

### 3.11. Role of SST on Brain Nitric Oxide Synthase Expression in SH-SY5Y Cells

In CNS, specifically, interneurons expressing SST-like immunoreactivity often co-expressed bNOS. Here, we determined whether bNOS-like immunoreactivity in non-differentiated and differentiated cells is under the influence of SST. bNOS expression was comparable to control in cells treated with SST (Figure 6C, panel a and b); whereas cells treated with RA and RA + SST displayed reduced bNOS-like immunoreactivity in the cell body but a strong localization in the neurites (Figure 6C, panels c and d). Furthermore, similar to immunocytochemistry observations results from immunoblot also showed a similar trend of bNOS expression displaying decreased expression in cell lysate prepared from RA and RA with SST treated cells (Figure 6D).

### 3.12. Developmental Changes in Glutamic Acid Decarboxylase-65 in SH-SY5Y Cells

We next determined the time-dependent distributional pattern of GAD-65 expression at days 3, 5, and 7 in control and RA treated SH-SY5Y cells by immunocytochemistry and immunoblot analysis. GAD-65-like immunoreactivity was well expressed in undifferentiated cells (Figure 7A, panels a–c). In comparison, cells treated with RA for days 3, 5, and 7 displayed reduced GAD-65-like immunoreactivity in the cell body but increased expression in neurites and apical ending of the cells (Figure 7A, panels d–f). Interestingly, GAD-65 immunoreactivity in differentiated cells is confined to the cell surface and neurite formation with a granular distribution pattern.

As shown in Figure 7B, undifferentiated SH-SY5Y cells showed a gradual increase in expression of GAD-65 from day 1 to 3 and remained comparable till day 7. Immunoblot analysis of GAD-65 in RA-treated SH-SY5Y cells peaked on day 3 with a reduced expression on days 5 and 7.

### 3.13. SST Regulates Glutamic Acid Decarboxylase-65 in SH-SY5Y Cells

To determine the presence of GABAergic phenotype in SH-SY5Y cells, GABA synthesizing enzyme GAD-65 activity was determined. Moderate GAD-65-like immunoreactivity was seen using immunocytochemistry or immunoblot when cells were treated with SST alone or in combination with RA (Figure 7C,D). In non-differentiated cells, GAD-65 immunoreactivity was widespread (Figure 7C, panel a) in contrast to the granular staining in cells treated with SST and RA alone or in combination (Figure 7C, panels b–d). In addition, GAD-65-like immunoreactivity was well expressed in neurite outgrowth of cells treated with RA and RA + SST (Figure 7C, panels c and d). With no significant difference, comparable expression of GAD-65 was observed using immunoblot analysis in control undifferentiated cells, SST, or RA treated cells (Figure 7D); however, the expression level of GAD-65 was reduced when SST was combined with RA.

### 3.14. GABA Immunoreactivity Suppressed in SH-SY5Y Cells during Differentiation

To determine whether changes in GAD have an impact on GABAergic neurotransmission in SH-SY5Y cells, GABA-like immunoreactivity was visualized on days 3, 5, and 7 by immunocytochemistry. GABA immunoreactivity was widely expressed intracellularly in undifferentiated SH-SY5Y cells at days 3–7 (Figure S1, panels A–C), with relatively strong expression at day 5 (Figure S1, panel B). Cells treated with RA displayed neurite formation with strong GABA staining at the apical ending of cells and neurites outgrowth (Figure S1, panels D–F). During differentiation, GABA-like immunoreactivity was significantly increased in neurites formation while diminished in the cell body. However, on day 7, GABA-like immunoreactivity decreased (Figure S1, panel F). These results indicate early expression of GABA, which declined gradually during the process of differentiation.

We also determined the GABA-like immunoreactivity in control and cells treated with SST, RA, and SST + RA. The localization of GABA-like immunoreactivity was confined to the cell surface as well as to neurites formation in cells treated with SST and RA alone or in combination (Figure S2).

## 4. Discussion

We recently demonstrated the neurite-promoting effect of SST in SH-SY5Y cells through association with MAP2, Tau and TUJ1 and signaling pathway extracellular signal-regulated kinase (ERK1/2) [10]. We also established an association between SST and TUJ1 in neurite formation of SH-SY5Y cells in response to RA [10]. Additionally, we described the role of SST in the regulation of collapsin response mediator protein 2, Ca^2+^ influx, cyclin-dependent kinase 5, calpain, and P35/25 in response to amyloid beta-induced toxicity [31]. These results implicate SST as a crucial player in the differentiation of SHSY-5Y cells and the formation of an intact and functional neuron. Yet, whether SST plays any role in regulating neurotransmitters phenotype in SH-SY5Y cells during RA-induced differentiation is unknown. Notably, the subcellular distribution, organization, and translocation to neurites formation is a critical determinant of neuronal maturity and functionality in differentiated SH-SY5Y cells. The present study first unraveled the developmental changes in SYP, SST, TH, ChAT, bNOS, GAD-65, and GABA-like immunoreactivity and expression during days 1–7 of differentiation. Second, we evaluated the role of SST alone or in combination with RA in the regulation of neurotransmitters expression and SYP translocation to neurites in differentiated SHY cells. Here, we present a distinct distribution pattern of different neurotransmitters in response to RA alone and in combination with SST in a time-dependent manner. To our knowledge, this is the first comprehensive characterization describing the developmental changes and the role of SST-mediated regulation of different neurotransmitter phenotypes in undifferentiated and RA-induced differentiated SH-SY5Y cells.

In a rather complicated but well-organized process of neuronal communication at the presynaptic site, the formation of synaptic vesicles and vesicle activation along with exocytosis and endocytosis are crucial determinants for functional neurons. SYP translocation to neurite during development is associated with the maturation of functionally active neurons as SYP is essential for neurotransmitters release [47,48]. Studies suggest weak expression of SYP in SH-SY5Y cells with restriction to soma before day 7 of differentiation and in neurite formation at a later point [47]. In contrast to these results, we found a gradual increase in SYP expression until day 5, which was suppressed at day 7 in differentiated cells, but not in undifferentiated cells. Moreover, SYP expression was two-fold higher at day 1 in the presence of RA when compared to non-differentiated SH-SY5Y cells. We also observed that SYP translocates along with the neurite in a time-dependent manner during differentiation. Increased expression of SYP in neurites following treatment with RA is consistent with previous observations [5]. Corroborating with our results, a restricted distributional pattern in the perinuclear region of the cells has also been reported for vesicle protein P38 and synaptic vesicle protein 2 in SH-SY5Y cells [49]. We observed a gradual suppression of SYP immunoreactivity in the perinuclear area, which increased in neurites when SST was added along with RA; however, interestingly, we observed that SYP expression was relatively low in RA alone and RA + SST treatment compared to undifferentiated SH-SY5Y cells. We speculate that the loss of SYP could be attributed to its translocation to the presynaptic terminals, which may include lysosomal degradation. Our speculation is further attested by studies reporting that RA increases lysosomal activity by enhancing the trafficking of enzymes from the TGN to late endosomes and lysosomes [50]. Thus, SST in SH-SY5Y cells might be associated with stabilization and migration of SYP, probably from TGN to the apical ending of the cell and eventually to neurites [47]. Moreover, SYP translocation to neurite formation might play a critical role in endocytosis and exocytosis of other crucial synaptic vesicle proteins and cannot be avoided from the discussion. Determination of molecular mechanisms associated with SYP processing from TGN to neurites is in progress.

SST plays a role as a trophic or apoptotic factor during embryonic brain development, thereby modulating neurogenesis, synaptogenesis, proliferation of cerebellar neuroblasts, and axonal pathfinding [51,52]. In developmental studies involving visual areas of rat and mouse cerebral cortex, SST neurons were visible from the first postnatal week, with a gradual increase in numbers up to about 3 weeks and thereafter reduced dramatically at later stages [53,54]. In parallel to these observations, we show a bimodal pattern of developmental changes in the expression of SST with a high expression on days 1 and 5, and a significant reduction on day 7. These results suggest that the SST is required during the early stage of differentiation. Furthermore, following SST treatment, a decrease in the intracellular SST expression attests that SST regulates its own release.

Here we speculate that RA indirectly enhances the expression of SST, which can be explained by the previous studies which demonstrated that RA increases the responsiveness of BDNF by inducing receptor tyrosine Kinase B [55]. Furthermore, BDNF is also known to enhance SST mRNA expression and its well-known role in neuronal development, survival, and differentiation [56]. Thus, it can be argued that RA enhances the expression of SST through increased responsiveness of BDNF in SH-SY5Y cells, thereby promoting neurite formation and regulating neurotransmitters.

The exact neuronal phenotype of SH-SY5Y cells following treatment with RA is not conclusive rather controversial. This is specifically true regarding TH expression. Lopes et al. reported increased expression of TH in SH-SY5Y cells differentiated with RA [24]; contrary to these observations, Cheung et al. demonstrated no changes in TH and emphasized that TH is already present in native SH-SY5Y cells [5]. In parallel to these contradicting results, we show time-dependent changes in TH-like immunoreactivity in response to RA. Additionally, consistent with previous studies, relatively low expression of TH in native cells support the presence of TH in undifferentiated cells. Korecka et al. described several positive and negative regulations of a transcriptional factor in SH-SY5Y cells in response to RA and described that RA induces DAergic-like phenotype in SH-SY5Y cells with increased DA levels and suppression of other neurotransmitters, including noradrenaline, glutamate, serotonin, and histamine [9]. RA is also involved in the stimulation of dopamine receptors [47]. Several previous in vitro and in vivo studies suggest that SST and DA regulate each other’s release and synthesis [57,58]. Furthermore, DA is involved in motor and psychological behavior, specifically by midbrain dopaminergic neurons, which are improved by SST [59]. Consistent with these previous findings, we observed enhanced expression of TH in response to SST alone and with RA supporting that DAergic system is well expressed in undifferentiated SH-SY5Y cells and enhanced in the presence of SST in combination with RA.

ACh plays a critical role during embryonic development, and decreased cholinergic transmission negatively impacts neurogenesis [25]. Furthermore, in vitro studies report that cholinergic stimulation improved the proliferation and survival of neural precursor cells [26,60]. Studies also suggest that ACh esterase inhibitor increases neural stem cell proliferation and promotes the survival of immature neurons [61]. Corroborating with these studies, increased expression of ChAT was seen in SH-SY5Y cells. Our results showed that SH-SY5Y cells expressed ChAT in a time-dependent manner and reached their peak on day 5 of differentiation, indicating that SH-SY5Y cells induce cholinergic transmission with RA treatment. It is known that cholinergic neurons inhibit SST release from neurons in the hypothalamus, while the release of the SST from dissociated cultured cortical neurons is stimulated by muscarinic agonists [62]. Our data showed that SST inhibits the expression of ChAT with or without RA; however, the immunoreactivity was more confined to neurite outgrowth, suggesting that SST might involve in the subcellular organization of ChAT in SH-SY5Y cells.

NO, synthesized from NOS, elicits various functions, including as a neurotransmitter, governs hormonal properties, cytoprotective, and cytotoxic molecule [63,64]. Fujibayashi et al. demonstrated that bNOS expression is crucial for RA receptor-mediated neurite growth and proposed association of kinases in SH-SY5Y cells [65]. Furthermore, enhanced bNOS has also been reported in other neuronal cells as well as in response to RA-differentiation [66,67]. Additionally, treatments with the NOS inhibitor or the NO scavenger hemoglobin increase cell proliferation and decrease neuronal differentiation [68]. Neurite formation in PC12 cells treated with NGF and angiotensin II treated NG108-15 cells involve NO in neurite outgrowth [69,70]. Our results partially support these previous studies. We found a significant increase in bNOS expression at day 1 with a gradual decline following differentiation until day 7. Conclusively, these results indicate that an early increase in bNOS is the prerequisite for neurite outgrowth and inhibition of cell proliferation in response to RA-induced differentiation. Furthermore, in the absence of NGF, NO alone has shown neurite outgrowth by implicating downstream signaling pathways, including ERK activation in PC12 cells [71]. Consistent with the observation that ERK activation is required for NO in promoting neurites formation, we have earlier reported changes in ERK following RA treatment [10]. In striatum and cortical brain regions, bNOS and SST are co-expressed and selectively spared in excitotoxicity and neurological diseases [72]. Our results suggest that SST treatment reduced the expression of bNOS in the cell body of undifferentiated and RA-induced differentiated SH-SY5Y cells; however, the expression of bNOS with SST + RA treatment was more and confined to neurites, suggesting the role of SST regulating bNOS during differentiation.

RA-induced differentiation of SH-SY5Y cells resulted in no significant changes in expression of GAD-65 and GABA in differentiated cells except a significant decrease at day 7. In contrast, a gradual increase in non-differentiated cells at days 3 and 5 was seen, followed by suppressed expression at day 7. The results described here implicate an inhibitory role of GABA in SH-SY5Y cells differentiation that might prompt the expression of other neurotransmitters and associated receptors; however, decreased expression in non-differentiated cells may allow cell proliferation and activation of cell survival pathways. Importantly, these observations warrant future studies to delineate the developmental changes in excitatory and inhibitory neurotransmission in SH-SY5Y cells. Previous studies have also described that a conditioned medium containing exosomes from GABA-treated Caco-2 cells activated SH-SY5Y cells [73]. In parallel to these observations, exosomes released from carnosine-treated Caco-2 cells are associated with neurite growth in SH-SY5Y cells [74]. Thus, we propose that higher expression of GABA and GAD in non-differentiated SH-SY5Y cells is a crucial factor in regulating the proliferation of cells. Furthermore, the colocalization of SST and GABA in the cerebral cortex suggests that SST presynaptically inhibits GABA release in a Ca^2+^-dependent manner [75]. Corroborating with these studies, the suppressed expression of GAD described here suggests that SST has a role in modulating inhibitory transmissions, thereby promoting neurite formations. Thus, SST acts to balance between crucial neurotransmitters and promotes neuritogenesis in SHSY-5Y cells.

## 5. Conclusions

Taken together, the results described here support the use of SH-SY5Y cells to delineate the role of SST in neuritogenesis with a possible implication in neurological diseases exhibiting interrupted neuronal communication, loss of cognitive function, and memory. We anticipate that SST maintains the inhibitory and excitatory balance and might stabilize cytoskeleton proteins during the process of differentiation, migration, and maturation of neurons. Whether this is a direct effect of SST via activating SST receptor (SSTR) subtypes or indirectly by regulating hormonal secretion or growth factors is unknown. GPCRs act via involving G proteins and are associated with signaling pathways linked to neurite formation and maturation. SSTR subtypes, a prominent member of the GPCR family, are responsible for multiple SST-mediated effects in CNS and peripheral tissues; however, which SSTR subtypes are associated with neurites formation and regulation of neurotransmitters in RA induced differentiation are not well defined, and further studies are in progress in this direction.

## Figures and Tables

**Table 1:** Semi-quantitative analysis of non-differentiated and RA-induced differentiated developmental changes in SH-SY5Y cells.

Markers	Conditions	Day 3	Day 5	Day 7
bNOS	ND	+++	+++	++
	D	+++	++	+
ChAT	ND	+++	+++	++
	D	+++	+++	++
GABA	ND	++	+++	++
	D	++	+++	+
GAD-65	ND	++	+++	++
	D	++	++	+
SST	ND	++	++	+
	D	+++	+++	+
SYP	ND	+	+++	++
	D	++	++	+
TH	ND	+++	++	+
	D	++	++	+

Relative values are ranked from +++: strongly positive; ++: moderately positive; +: weakly positive. ND—Non-differentiated; D—Differentiated cells; bNOS—brain nitric oxide synthase; ChAT—Choline acetyltransferase; GABA—Gamma-aminobutyric Acid; GAD-65—Glutamic acid decarboxylase-65; SST—Somatostatin; SYP—Synaptophysin; TH—tyrosine hydroxylase.

## Data Availability

Data is contained within the article or supplementary material.

## Supplementary Materials

The following are available online at www.mdpi.com/article/10.3390/biomedicines10020337/s1, Figure S1: Representative photomicrographs illustrating GABA immunoreactivity in undifferentiated and differentiated SH-SY5Y cells. Figure S2: GABA expression in SH-SY5Y cells is regulated by SST.
