# Supplementary material for: Somatostatin-Mediated Regulation of Retinoic Acid-Induced Differentiation of SH-SY5Y Cells: Neurotransmitters Phenotype Characterization
Source: Biomedicines. 2022 Feb 1;10(2):337. doi: 10.3390/biomedicines10020337 (PMC8961784; doi:10.3390/biomedicines10020337)
Supplement: Supplementary file 1 [file biomedicines-10-00337-s001.zip › biomedicines-1525349-supplementary.pdf]

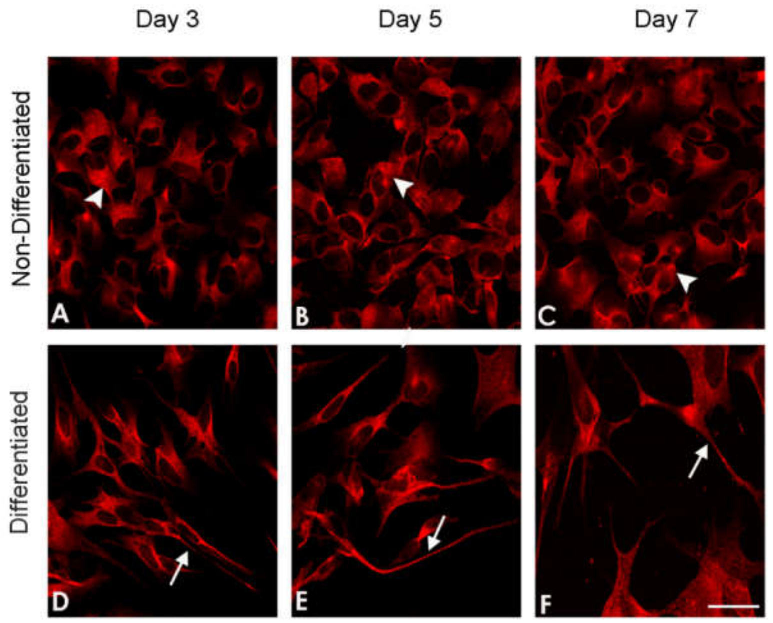

Figure S1: Representative photomicrographs illustrating GABA immunoreactivity in undifferentiated and differentiated SH-SY5Y cells. In undifferentiated SH-SY5Y cells (**A-C**), GABA expression was confined intracellularly (arrowhead) with increased staining on day 5 (**B**). In the presence of RA, the expression of GABA reduced in the cell body and strong immunoreactivity was seen in apical endings and neurites (arrow) till day 5 of differentiation (**D and E**). GABA-like immunoreactivity in neurites and intracellularly was reduced on day 7 with RA treatment (**F**). Scale bar = 20μm.

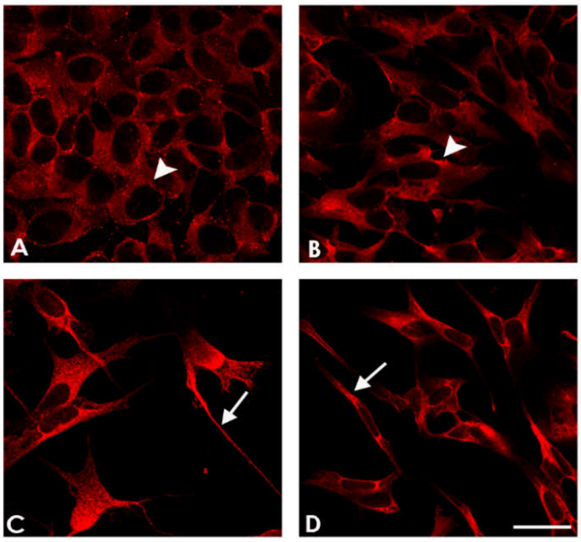

Figure S2: GABA expression in SH-SY5Y cells is regulated by SST**.** GABA-like immunoreactivity was well expressed in the cell body of control (**A**), and SST (**B**) treated cells (arrowhead). GABA-like immunoreactivity moved towards neurites formation (arrow) in cells treated with RA alone (**C**) or in combination with SST (**D**). Note: Immunoreactivity of GABA was reduced in cells treated with SST+RA compared to RA alone. Scale bar = 20μm.
